# Characteristics and outcome of influenza-associated encephalopathy/encephalitis among children in a tertiary pediatric hospital in Italy, 2017–2019

**DOI:** 10.1186/s12879-019-4636-5

**Published:** 2019-11-29

**Authors:** Maria Vincenza Mastrolia, Chiara Rubino, Massimo Resti, Sandra Trapani, Luisa Galli

**Affiliations:** 1 Post-graduate School of Pediatrics, University of Florence, Viale Gaetano Pieraccini, 24, 50139 Florence, Italy; 2 Pediatric Department, Anna Meyer Children’s University Hospital, Florence, Italy; 3 Department of Health Sciences University of Florence, Paediatric Infectious Diseases Division, Anna Meyer Children’s University Hospital, Florence, Italy

**Keywords:** Influenza, Children, Encephalopathy, Encephalitis, Clinical characteristics, Outcome

## Abstract

**Background:**

Influenza is the most frequent cause of acute upper respiratory tract infections during winter season. Although rare, neurological manifestations are known to occur during influenza infection and approximatively three-quarters of cases are in children. In this study, we aimed to characterize the burden and clinical spectrum of influenza-associated encephalopathy and encephalitis in children admitted at a tertiary pediatric hospital in Italy over two influenza seasons (2017–2019).

**Methods:**

We retrospectively analyzed clinical, laboratory, instrumental data and outcome of patients discharged with ICD9-CM 487.0 code.

**Results:**

Fifteen children (13.1% of those discharged with a diagnosis of influenza infection in the study period), had influenza-associated central nervous system (CNS) manifestations. Eight patients (53.3%) were diagnosed as influenza encephalitis, 7 (46.7%) as influenza encephalopathy. Median age was 27 months. In children under 2 years of age (40% of all cases) altered consciousness was the most frequent neurological manifestation while respiratory symptoms were present at admission in all cases. Younger children also required intensive care support more frequently. Five subjects (33.3%) presented comorbidity. None of the patients had received seasonal influenza vaccination. The median time from onset of respiratory signs to onset of neurological manifestations was 24 h. Cerebrospinal fluid (CSF) analysis was normal in most patients and polymerase chain reaction for influenza virus RNA on CSF, when performed, was negative in all samples. Neuroradiological investigations, performed in 5 children, reported cortical and subcortical white matter signal alterations. Oseltamivir was administered only in 2 cases. Fourteen patients recovered without sequelae, and only a 2-year-old girl had minimal impairment in fine motor skills at discharge.

**Conclusions:**

All children presenting acute neurological features during influenza season should be evaluated for influenza-associated CNS complications even if the respiratory involvement is mild. Absence of underlying diseases or other risk factors are not protective factors against CNS influenza-associated complications. The lack of CSF pleocytosis does not exclude CNS involvement. Children under 2 years of age are at higher risk of requiring intensive care support.

## Background

Influenza primarily affects the respiratory system and represents one of the most frequent causes of acute upper respiratory tract infections during the winter season. Signs/symptoms of influenza infection usually include fever, headache, cough, sore throat, myalgia and sometimes diarrhea and vomiting. The infection is usually self-limiting, although children, elderly people, immunocompromised patients and pregnant women have a higher risk of complications [1]. Central nervous system (CNS) involvement is rare, but is an important complication of influenza infection with approximatively three-quarters of cases regarding children [2]. Neurological complications are reported to occur in 1–15% of influenza cases in the pediatric age, often self-resolving, although permanent sequelae or death can occur [2, 3].

Influenza accounts for 2–11% of cases in studies of childhood encephalitis. Moreover, [2, 4–8] in the US, the rate of influenza-related neurological complications has been estimated to be 4 per 100,000 children per year. The mean annual incidence in Australian children is 2.8 per 1,000,000 population [2]. In a Canadian study, encephalitis was documented in 1.9% of children hospitalized for influenza over eight non-pandemic seasons from 2004 to 2013 [7].

A comorbidity or a preexisting disease, especially a neurodevelopmental disorder, is associated to increased risk of neurological influenza-associated complications [2–4].

The neurological disease is often defined noted as influenza-associated encephalitis/encephalopathy (IAE). The term encephalopathy is preferred because influenza virus is rarely neuroinvasive, although a small number of published cases have been described with detection of influenza virus RNA in the cerebrospinal fluid (CSF) by means of molecular methods [3, 9]. IAE may include a variety of clinical and radiological syndromes frequently associated with viral infections, including influenza viruses [4]. Cases of IAE have been reported since the 1990s in the Asian continent, especially in Japan. During the 2009 H1N1 pandemic, IAE cases were frequently reported worldwide [10].

Neurological sign/symptoms attributed to influenza range from a mildly altered mental state, vertigo and brief febrile seizures to life threatening complications such as status epilepticus, meningitis, stroke, and demyelinating disease [6]. Antiviral agents as neuraminidase inhibitors, and immunomodulatory treatments (corticosteroids, intravenous immunoglobulin), are currently administered but the evidence on their efficacy is limited. At the moment, there are no available biomarkers to predict outcome. Some neuroradiological features, in particular extensive changes on magnetic resonance imaging (MRI), are reported to be related to disease severity [10–14].

Available literature data about neurological manifestations of influenza in children is limited, and only single case reports or small case series on this issue are available [6–8, 10, 15, 16].

In this study, we aimed to characterize the burden and clinical spectrum of influenza-associated neurological disease in children admitted to a tertiary pediatric University- Hospital in Italy over two influenza seasons (2017–2019).

## Methods

### Study population

We retrospectively identified children aged between 1 month and 14 years with evidence of influenza and associated neurological disease admitted to Anna Meyer Children’s University Hospital during two influenza seasons, between October 2017 and April 2019.

Eligible patients were retrospectively searched in the regional hospital discharge database using the International Classification of Disease, Ninth Revision, Clinical Modification (ICD-9-CM) 487.0 code.

The project has been approved by the Ethics Committee of the “Azienda Ospedaliero-Universitaria Meyer” of Florence on March 2017 (Hospital Institution Review Board Cod 87).

Confirmed influenza infection was defined as detection of influenza virus RNA by polymerase chain reaction (PCR) in nasal swabs, respiratory samples or CSF. Either children cases with CNS signs/symptoms as presenting features or those with subsequent complications were included. Children were excluded when influenza was not confirmed, hospital admission was not required and when an alternative cause could better explain neurological manifestations.

Moreover, in order to evaluate age-related clinical and laboratory differences, two groups (≤2 and > 2 years of age) were considered.

Statistical analysis was performed using SPSS (Version 25.0, SPSS, Inc., Chicago, IL, USA). Metric data were tested for normal distribution. Continuous variables were expressed as median values and interquartile ranges (IQRs) and compared by means of Mann–Whitney U test. The chi-square test or Fisher’s test, when appropriate, were performed to evaluate differences between categorical variables. *P* <  0.05 was considered statistically significant.

### Data collection

For each patient, the following data were collected: demographic and clinical characteristics, influenza vaccination status, pediatric intensive care unit (PICU) admission and length of stay, laboratory results, including complete blood count, acute phase reactants (C reactive protein and procalcitonin values), serum and CSF biochemistry, instrumental findings in computed tomography (CT) or MRI, electroencephalography (EEG) findings, treatments and procedures adopted during the hospitalization and short-term outcome. Patient data was obtained from hospital computer database system and patients’ medical records. Brain CT and MRI were evaluated by a neuroradiologist who had knowledge of the patients’ clinical information. Main radiological evaluation included locations with abnormal signal, signal alteration and enhancement.

### Clinical case and outcome definitions

Clinical case definition was adapted from those used for CNS infections based on the clinical features, CSF and neuroimaging results [7].

An influenza associated neurological complication was defined as the occurrence or worsening of neurological involvement, which could not be explained otherwise, in a patient with laboratory-proven influenza. Complications included central or peripheral nervous system involvement, such as seizures, encephalopathy, encephalitis, Guillain-Barre syndrome, acute disseminated encephalomyelitis (ADEM), ataxia, involuntary movements, and behavioral or personality disorders .

Encephalopathy was defined as the presence of altered mental status including behavioral changes with no evidence of inflammation of the CNS [7].

Encephalitis was defined as encephalopathy accompanied by two or more of the following findings: a fever or history of fever, seizure or focal neurological finding, CSF pleocytosis, EEG alterations or abnormal neuroradiological findings indicative of infection or inflammation [7].

Meningitis was defined as CSF pleocytosis associated to meningeal symptoms (headache, nuchal rigidity and altered state of consciousness) [7].

The normal ranges of white blood cells (WBC), neutrophil and platelet count were defined 5000-13,000/mm^3^, 1500-7000/mm^3^ and 150,000-400,000/mm^3^, respectively.

The short-term outcome was classified in four categories: recovery without sequelae (good prognosis), minimal or mild sequelae that did not interfere with daily life, serious sequelae that resulted in need for assistance in daily life or the worsening of existing neurological state and death.

## Results

Fifteen patients with influenza-associated neurological manifestations were enrolled, representing 13.1% of all children discharged with influenza diagnosis in the study period.

Eight patients (53.3%) were diagnosed as influenza encephalitis, 7 (46.7%) as influenza encephalopathy. No cases of meningitis or peripheral nervous system involvement were detected.

Table 1 shows the demographic and clinical characteristics of our patients. Median age was 27 months (IQR: 7–48) and 40% of children were under 2 years of age. Eleven patients (73.3%) were female. Five subjects (33.3%) presented comorbidity: four had neurological underlying diseases (ventriculomegaly, epileptic encephalopathy, epilepsy and Dravet syndrome) and one had congenital heart disease. None of the patients had received seasonal influenza vaccination. Time between the onset of flu-like symptoms and neurological manifestations was 24 h. Fourteen patients were admitted to the Pediatrics department, one patient was firstly admitted to PICU. Fever was present in 13 patients at admission. The spectrum of neurological manifestations included altered consciousness in 8 children (53.3%), seizures in 6 (40%), ataxia in 1 (6.7%). Most of the children (73.3%) presented respiratory symptoms at admission (4 patients complained of dyspnea, seven had cough).

**Table 1:** Demographic and clinical characteristic of patients

Age (month) [median (IQR)]: 27 (7–48)	
0- ≤ 2 years [n (%)]:	6 (40%)
2–10 years [n (%)]:	9 (60%)
Sex	
Male [n (%)]:	4 (26.7%)
Female [n (%)]:	11 (73.3%)
Comorbid disease [n (%)]:	5 (33.3%)
Chronic neurological diseases: [n (%)]:	4 (26.7%)
Congenital heart disease [n (%)]:	1 (6.7%)
Seasonal influenza vaccination:	none
Time between flu-like symptoms and neurological manifestations	
[median (IQR)]:	1 day (0 h-3 days)
Unit or department of admission [n (%)]	
Pediatric department:	14 (93.3%)
Pediatric intensive care unit:	1 (6.7%)
Duration of hospitalization	
[median (IQR)]:	9 days (7–12 days)
Clinical presentation at admission [n (%)]	
Fever:	13 (86.7%)
• Maximum [median (IQR)]	38.7 (37.6–39.2) °C
Neurologic manifestations:	15 (100%)
• Seizures:	6 (40%)
• Altered consciousness:	8 (53.3%)
• Ataxia:	1 (6.7%)
Respiratory manifestations:	11 (73.3%)
• Cough:	7 (46.7%)
• Dyspnea	4 (26.7%)
Other influenza-related complications [n (%)]	
Respiratory failure:	4 (26.7%)
Liver impairment:	1 (6.7%)
PICU admission:	4 (26.7%)
Days in PICU [median (IQR)]:	5.5 (4.25–15)

Four subjects (26.7%) developed respiratory failure and required PICU admission. One of these patients also experienced liver impairment. The median length of stay in PICU was 5.5 days.

Laboratory values at admission are summarized in Table 2. Median leukocyte count was 8050 cells/mm3 (IQR: 6710-12,790). One patient had leukopenia (WBC < 5000/mm^3^) and 3 cases showed leukocytosis. Neutrophil count was elevated in 7 cases (46.7%) and lymphocyte count was decreased in 8 cases (53.3%), none of the patients had neutropenia or lymphocytosis. Median platelet count was 288,000 cells/mm^3^. Two children showed thrombocytopenia; thrombocytosis was observed only in one subject. Alanine aminotransferase (ALT) median level was 26 UI/L, with 2 children presenting a minimal elevation with maximum value of 65 UI/L. C reactive protein (CRP) median level was 1.78 mg/dL and 3 children presented normal values. Procalcitonin was performed in 7 patients at admission: its median value was 1.2 ng/mL and it was normal (< 0.5 ng/mL) in 3 children.

**Table 2:** Analysis of blood laboratory findings at admission

Laboratory finding	median value (IQR)	normal n (%)
Leucocyte count (/mm^3^) *normal range: 5000-13,000*	8,050 (6,710-12,790)	11 (73.3%)
Neutrophil count (/mm^3^) *normal range: 1500-7000*	6,031 (2,912-10,215)	8 (53.3%)
Lymphocyte count (/mm^3^) *normal range: 1500-4000*	1,356 (808–2,212)	7 (46.7%)
Platelet count (/mm3) *normal range: 150,000-400,000*	288,000 (237,000-443,000)	12 (80%)
Sodium (mEq/L) *normal range: 135–145*	136 (133–138)	9 (60%)
ALT (U/L) *normal range: 8–45*	26 (21–41)	13 (86.7%)
CRP (mg/dL) *Normal range: < 0.29*	1.78 (0.61–2.46)	3 (20%)
Procalcitonin (ng/mL) *normal range: < 0.5*	1.2 (0.5–2.1)	3 (42.9%)

*Legend: For each parameter we report median value (IQR), number and percentage of normal values in the case series. These data refer to blood samples which were collected within six hours of hospital admission. Laboratory data at admission were available for all the 15 patients, except for Procalcitonin, which was performed in 7 patients at admission*

Table 3 summarizes clinical and laboratory differences between the two age groups 0- ≤ 2 years and 3–10 years. In the first group, all patients had altered consciousness as the main neurological manifestation. On the contrary, older children more frequently manifested seizures at onset (6 patients), whereas altered consciousness and ataxia were reported in 2 subjects and 1 subject, respectively. Respiratory symptoms developed in all children under 2 years of age compared to 5 of the 9 older patients. Laboratory values and clinical signs/symptoms did not show significant differences according to age. However, all children with respiratory failure and liver impairment were in the younger group (*p value* 0.011).

**Table 3:** Clinical and laboratory difference in age subgroups

	0- ≤ 2 years: 6 (40%)	3–10 years: 9 (60%)	p value
Neurological manifestation			
-Altered consciousness	5	4	0.287
-Convulsions	0	6	0.028
Respiratory manifestations	6	5	
-Dyspnea	3	1	0.235
Laboratory values			
-leukocyte count (/mm^3^)	8,835	7,290	1.000
-neutrophil count (/mm^3^)	6,564.5	6,031	0.607
-lymphocyte count (/mm^3^)	1,928.5	1,093	0.776
-Sodium level (mEq/L)	136	135	0.776
-CRP value at admission (mg/dl)	1.9	2.38	0.088
-Highest CRP value (mg/dl)	2.2	2.46	0.955
Other influenza-related complications			
-PICU admission	4	0	0.011

*Legend: We analyzed difference in two age subgroups: 0–2 years, comprising 6 patients (40% of the sample) and 3–10 years, comprising 9 patients (60% of the sample). For each neurological manifestation, respiratory manifestation and other influenza-related complication, we reported the number of patients in the subgroups presenting the feature and searched for a statistically significant difference (p < 0.05). For each laboratory value, we reported median values in the subgroups and searched for a statistically significant difference(p < 0.05)*

PCR assays on throat or nasal swab detected influenza A in 93.3% of patients, with H1N1 subtype found in 12 cases (80%) and H3N2 in 2 children (13.3%). One child (6.7%) had influenza B.

Lumbar puncture was performed in 7 patients (46.7%). CSF analysis showed pleocytosis and elevated protein levels only in 1 child; another subject presented mildly elevated protein levels without any other abnormalities. PCR for influenza RNA on CSF was performed in 4 patients (26.7%) resulting negative.

EEG was performed in eleven patients (73.3%). EEG records revealed generalized slowing in four patients, focal slow wave activity in one patient. Two children showed findings consistent with epileptic encephalopathy and Dravet syndrome, respectively.

Neuroimaging was performed in five subjects (33.3%): four children had both CT and MRI, one patient had only CT images. CT showed hypodensity of cerebral white matter at the vertex only in one case. Brain MRI detected non-specific abnormalities in all children investigated, but in 1 of the 4 cases MRI alterations were associated with neurological underlying diseases. In patient #9, it showed mild expansion of some perivascular spaces in the periventricular white matter. In patient #11, MRI showed known alterations of Dravet syndrome. In patient #12, it revealed DWI (diffusion weighted imaging)-hyperintense and ADC (apparent diffusion coefficient)-hypointense cortical and subcortical areas: these findings were consistent with encephalitis. In patient #15, some diffusion-restricted, non-contrast-enhanced areas in the periventricular white matter and semi oval center were found: these findings were consistent with ischemic areas in the context of an inflammatory/infectious process.

Only 2 patients (13.3%) were treated with oseltamivir during PICU hospitalization. Five children (33.3%) received steroid therapy with dexamethasone. No other adjunctive treatments such as immunoglobulins were administered.

Fourteen patients recovered without sequelae. Only a 2-year-old girl showed minimal impairment in fine motor skills at the moment of discharge.

Table 4 summarizes influenza typing, findings of CSF, EEG, neuroimaging, case definition, treatment and outcome in all case series.

**Table 4:** Influenza typing, CSF analysis, neuroradiological imaging, clinical case definition, comorbidity, treatment and outcome

Patient (n)	Age (months)	Influenza virus subtype	CSF analysis	EEG pattern	Brain CT/MRI	Diagnosis	Comorbidity	Oseltamivir	Steroids	Outcome
1	7	H3N2	Normal	Generalized slowing	Not performed	Encephalitis	no	yes	no	Recovery without sequelae
2	1	H1N1	pleocytosis, elevated protein levels	Not performed	Not performed	Encephalitis	pulmonary stenosis	no	no	Recovery without sequelae
3	7	H1N1	Normal	Generalized slowing	Not performed	Encephalitis	ventriculomegaly	yes	yes	Recovery without sequelae
4	20	H1N1	Not performed	Not performed	Not performed	Encephalopathy	no	no	No	Recovery without sequelae
5	14	H1N1	Not performed	Not performed	Not performed	Encephalopathy	no	no	No	Recovery without sequelae
6	38	H1N1	Not performed	Generalized slowing, focal abnormalities	Not performed	Encephalitis	No	no	No	Recovery without sequelae
7	27	H1N1	Not performed	Normal findings	Not performed	Encephalopathy	epileptic encephalopathy	no	No	Recovery without sequelae
8	120	H1N1	Not performed	Normal findings	Not performed	Encephalopathy	no	no	no	Recovery without sequelae
9	72	H3N2	Normal findings	Generalized slowing	CT: normal findings MRI: expansion of some perivascular spaces in periventricular white matter	Encephalitis	epilepsy	no	yes	Recovery without sequelae
10	6	H1N1	Normal findings	Normal findings	CT: Normal findings MRI: not performed	Encephalopathy	No	no	no	Recovery without sequelae
11	30	H1N1	Not performed	Consistent with comorbidity (Dravet syndrome)	CT: Normal findings MRI: consistent with comorbidity (Dravet syndrome)	Encephalopathy	No	no	yes	Recovery without sequelae
12	25	H1N1	Normal findings	Normal findings	CT: Normal findings MRI: cortical and subcortical areas DWI-hyperintense and ADC hypointense	Encephalitis	Dravet syndrome	no	yes	Minimal sequelae
13	48	H1N1	Not performed	Not performed	Not performed	Encephalopathy	no	no	no	Recovery without sequelae
14	72	B	Not performed	Generalized slowing	Not performed	Encephalitis	No	no	no	Recovery without sequelae
15	48	H1N1	Mildly elevated protein levels	Focal slow wave activity	CT: Hypodensity of cerebral white matter at the vertex MRI: diffusion-restricted areas in periventricular white matter and semioval center	Encephalitis	No	no	yes	Recovery without sequelae

## Discussion

Influenza is a common infectious agent worldwide. It primarily affects the respiratory system. However, in some cases, neurological involvement may be predominant or even the presenting finding. In our case series, we reviewed the clinical and laboratory data, radiological features and outcome in 15 children hospitalized in a tertiary pediatric University-Hospital, presenting neurological manifestations during influenza infection, over two influenza seasons (2017–2019).

In the literature, the reported frequency of neurological complications in hospitalized children with influenza infection ranges from 1.7 to 15% in different studies [6]. In our retrospective analysis, we observed 114 cases of influenza infection requiring hospitalization during two influenza seasons and, among these patients, 15 children experienced neurological symptoms resulting in a proportion of 13.1%, as already reported [2, 6–8, 10, 15].

About half of patients with neurological involvement (53.3%) in our case series were diagnosed as influenza encephalitis, and half (46.7%) as influenza encephalopathy.

Moreover, we found that young age (i.e. younger than 2 years), as already reported, is an important risk factor for the occurrence of influenza-associated neurological complications [16].

In addition, younger children more frequently require intensive care support: we reported 4 cases that needed PICU admission for respiratory failure and liver impairment and all of them were children younger than 2 years.

A systematic review by Nair et al. estimated that, worldwide, the number of new influenza episodes, influenza-associated acute lower respiratory infections (ALRI), and severe influenza-associated ALRI in children younger than 5 years amounted to 90 million, 20 million (13% of all cases of pediatric ALRI), and 1 million (7% of cases of all severe pediatric ALRI) respectively in 2008. Considering this age group, 28,000–111,500 deaths were attributable to influenza-associated ALRI, and almost all of them (99%) occurred in developing countries. Implementation of influenza vaccine administration, especially in children under 2 years of age, could substantially reduce sequelae and mortality associated with this disease [17].

Moreover, our study reports that one-third of children had a comorbidity or a preexisting disease, especially a neurodevelopmental disorder, suggesting a possible association with increased risk of complications. In particular, neurologic disorders are considered a strong risk factor for influenza-related neurologic complications such as seizures, IAE or cerebral infarction. Although they constitute a small category of the pediatric population, children with neurologic disorders represent a significant fraction of patients hospitalized and died as a consequence of influenza-associated complications [18].

None of our children had been vaccinated for seasonal influenza, consistent with other studies [2, 6–8, 19]. It is not proven that vaccination has a preventable role against neurological manifestation and a study to demonstrate vaccine effectiveness appears impractical, due to low case numbers. However, one study from Japan showed that the mortality rate from IAE in children was significantly lower during the era of mass influenza immunization (pre-1994) than in a subsequent era (1995–2000) [20].

Therefore, influenza vaccination, which is recommended in young children and strongly recommended in children with underlying diseases, could avoid influenza-associated complications in our children.

Most of our patients were previously healthy. This data, in agreement with prior studies [21–23], confirms that a history of good health is not a protective factor against the development of influenza-associated neurological complications.

Our case series reports that most cases were influenza A infection (93.3%) with 80% of total amount attributed to H1N1 virus and only one case of influenza B infection in a 6-year-old child. In 2017–18 season, virus B was dominant in influenza season in Italy, causing 60% of influenza cases. H1N1 caused 36% of cases, H3N2 caused 2% of cases; the remaining 2% were not subtyped virus A cases [24]. In 2018–19 in Italy, H1N1 caused 46% of cases, H3N2 caused 46% of cases; the remaining 8% were not subtyped virus A cases. The percentage of virus B cases was 0% [25]. Therefore, different circulation of influenza B in the two last influenza seasons could explain that the only case of influenza B in our paper was reported during 2017–18 season.Available data from literature show that the majority of cases of IAE are due to influenza A infection, while influenza B is responsible for around 10% of cases [26]Similarly to our paper, Paksu et al. [6] describe a higher prevalence of H1N1 virus in children presenting IAE. Therefore, both the epidemiology of virus circulation and the frequent association of virus A, particularly H1N1, to IAE could explain the virological distribution of our case series. In addition, Wada et al. [16] reported that influenza virus shows an age distribution with higher incidence of type B in older children. This correlation is maybe related to a small prevalence rate of influenza B antibody at all ages because of the small epidemics scale of type B virus. In our series, the patients’ young median age may explain the low prevalence of type B virus infection.

Neurological complications may present early or late in the course of the infection. Our case series had a short (24 h) time from onset of fever/respiratory signs to neurological complications. This is in agreement with most of the available data which reported an interval shorter than 48 h between the occurrence of fever and the appearance of neurological findings [6–8, 19].

In accordance with literature, our study confirms that altered consciousness and seizures were the most frequently reported neurological findings [6].

In our experience, all the patients under the age of two presented respiratory symptoms at the admission and the only reported neurological manifestation was altered consciousness. These data differ from a previous study which observed convulsions as the initial neurological sign in children younger than 5 years [16].

In our study, laboratory data did not show any significant alteration either when the whole study group was observed, or when the cohort was divided into the two different age groups, as reported in available literature [2, 5–8, 19, 27].

CSF analysis, performed in 7 patients, showed pleocytosis and elevated protein levels only in one case. Accordingly, previous studies report a low rate of CSF pleocytosis in most of the patients with clinical and neuroradiological evidence of central nervous system involvement [2, 6–8]. This finding suggests that nervous system injury may be related to inflammatory or immune-mediated response [28–30]..

The mechanism underlying the development of influenza-associated neurological complications is not clear. Some patients with specific genetic backgrounds, infected by certain influenza strains, release proinflammatory cytokines that induce vascular endothelial injury and increase blood brain barrier (BBB) permeability [1].

In some patients, viruses could infect the CNS through the peripheral nerve or damaged BBB inducing a CNS cytokine storm, with direct damage and promotion of apoptosis of neurons and glial cells. Whether the influenza virus invades the CNS or not is still controversial [1]. Only a small number of patients were positive for viral RNA in the CSF and brain in available studies [9]. In our case series, PCR for influenza virus RNA on CSF resulted negative in all patients in which it was performed, as in the majority of previous reports [1, 2, 4, 6–8].

To date, there are no biochemical markers that can help predicting clinical course and prognosis of neurological complications during influenza. Three recent studies proposed that the dosage of specific molecules in patients’ serum or CSF may help the diagnosis and predict the outcome. Morichi et al. have found increased CSF vascular endothelial growth factor and platelet derived growth factor (PDGF) in IAE and bacterial meningitis (BM) patients in comparison with a control group. Since cerebrospinal fluid PDGF was more markedly increased in the IAE than in the BM group, vascular endothelial disorder may be more severe in IAE and it may be useful for differential diagnosis [31]. Macdonald-Laurs et al. have measured CSF neopterin levels in patients with neuro-influenza. Neopterin is a biomarker of inflammation synthesized by human macrophages, it is a catabolic product of guanosine triphosphate. Neopterin levels were elevated in the majority of tested children (8/10) with encephalopathy, suggesting a potential use as a diagnostic biomarker [11]. Sun et al. showed that serum neutrophil elastase was significantly elevated in IAE compared with uncomplicated influenza [32].

Neuroradiological studies may assist in confirming the diagnosis, evaluating the severity of clinical state and guiding decisions regarding treatment. In our patients’ group, a neuroradiological study was performed in 5 children. The only reported neuroradiological abnormalities were cortical and subcortical white matter signal alterations.

The manifestations and neurological outcomes of IAE vary across countries. Currently, there is no recommendation for standard of care, but anti-viral medications against influenza could be beneficial. The Infectious Diseases Society of America guidelines suggest that oseltamivir be considered in case of influenza related neurological complications [33]. Although there is very limited evidence for a benefit, given the severity of disease, the timing of neurologic symptoms and the likely role of infection in triggering CNS inflammation, an empiric use of oseltamivir during influenza season may be considered [2, 4]. In contrast to previous studies, this drug was infrequently administered in our patients [7]. On the contrary, five patients received steroid treatment. In literature, role of steroid pulse therapy and immunoglobulin is still controversial. However, they are frequently used in IAE because an immune-mediated mechanism may be a major pathogenetic component [19].

As regards outcome, 14 patients recovered without sequelae, and only a 2-year-old girl experienced a minimal impairment in fine motor skills at the moment of discharge. Conversely, previous reports have highlighted a high frequency of poor outcomes with deaths and significant neurologic sequelae at discharge in the survivors [2, 6–8, 19]. In literature, adverse short-term outcomes were associated with specific acute encephalopathy syndromes: the majority of deaths occurred in children with acute necrotizing encephalopathy (ANE) [7]. ANE was not described in any of our patients. The virus subtypes and patients’ characteristics (median age, comorbidity status, vaccination status, history of good health, time of onset of neurological manifestations) of our case series do not significantly differ from available literature studies. We cannot compare our data to other regional or national data because there are no other Italian studies about IAE. On the other hand, the majority of available studies regard Asiatic case series [3, 4, 9, 10, 19]. It is thus possible that genetic factors could affect the risk of developing ANE and mortality related to IAE. Moreover, our patients more frequently received steroid treatment if compared to available literature studies. Further studies are needed to establish if steroid treatment has a protective effect on mortality and short-term outcomes.

Our study presents several limitations. Primarily, it is a single center study and collection of data was retrospective. We analyzed a relatively small number of children; moreover, laboratory and radiological investigations were not performed in all patients. Finally, we did not collect data for a long-term evaluation.

## Conclusions

In conclusion, influenza-associated neurological complications are frequent in children.

All children who are admitted with neurological findings during the influenza season should be evaluated for influenza-associated neurological complications even if their respiratory complaints are mild or nonexistent. A history of good health is not a protective factor against the development of neurological symptoms. Children under 2 years of age more frequently require intensive care support. The lack of CSF pleocytosis does not exclude nervous system involvement. Given the reported correlation of clinical and MRI findings with prognosis, the recourse to neuroimaging is necessary. During influenza season, empirical use of oseltamivir should be considered in case of neurological complications. In our cohort, none of the patients was vaccinated, although 14 out of 15 were eligible for vaccination. Considering the cost-effectiveness of vaccination if compared to influenza-related hospitalization, we recommend influenza seasonal vaccination for all eligible children, and particularly for children with underlying diseases.

Further studies with a larger population are needed for better understanding of prevalence and pathogenesis of IAE, for detection of biomarkers of neurological involvement and disease severity and for investigation of long-term outcome.

## Data Availability

The datasets used and analyzed during the current study are available from the corresponding author on reasonable request.

## Abbreviations

ADEM: Acute disseminated encephalomyelitis
ALRI: Acute lower respiratory infections
ALT: Alanine aminotransferase
ANE: Acute necrotizing encephalopathy
BBB: Blood brain barrier
BM: Bacterial meningitis
CNS: Central nervous system
CRP: C reactive protein
CSF: Cerebrospinal fluid
CT: Computed tomography
EEG: Electroencephalography
IAE: Influenza-associated encephalitis/encephalopathy
IQR: Interquartile range
MRI: Magnetic resonance imaging
PCR: Polymerase chain reaction
PDGF: Platelet derived growth factor
PICU: Pediatric intensive care unit
WBC: White blood cells
